# P292: HCV transmission in a gastroenterology endoscopy unit

**DOI:** 10.1186/2047-2994-2-S1-P292

**Published:** 2013-06-20

**Authors:** M Cantero Caballero, P Menchen, A Avellon, C Fiqueroa, MJ Ruano, P Rodriguez

**Affiliations:** 1 Department of Preventive Medicine and Quality Management, Madrid, Spain; 2 Gastrointestinal Endoscopy Unit, General University Hospital Gregorio Marañón, Madrid, Spain; 3 Área de Virología, Centro Nacional de Microbiología Instituto de Salud Carlos III, Madrid, Spain

## Introduction

HCV Transmission in health-care settings is an increasingly recognized public health problem. There are few documented cases of transmission during endoscopy.

## Objectives

We report a case of HCV transmission during an upper digestive endoscopy.

## Methods

In January 2012, the case patient contacted the Gastroenterology Endoscopy Unit(GEU) as he believed that he had acquired HCV during an outpatient endoscopy procedure in December 2011. Preventive Medicine Unit performed an assessment of the infection control practices. We also reviewed clinical and laboratory data from the case patient as well as from patients who underwent endoscopy on the same day. Laboratory tests included total and specific HCV antibodies (Ab), HCV IgG Ab avidity test specific IgG Ab and aNS5B(500 nt) fragment phylogenetic comparison with the Neighbor-Joining method, 1000 repeats, bootstrap analysis including 188 sequences for comparison.

## Results

Review of the case patient medical record revealed negative Ab 10 days prior to the onset of symptoms, being positive (total HCVAb and anti NS3, low avidity) after 13 days. A positive HCV patient with the same genotype(1b), who underwent upper endoscopy before the case patient, was identified during the review of the GEU medical appointments. The same endoscope was used in both patients. Blood samples from both patients were sent to the national hepatitis laboratory. The putative source patient presented high HCVAb avidity, chronic infection. Phylogenetic analysis revealed that sequences obtained clustered in a monophyletic group (bootstrap value 95%). Endoscopy technique and endoscope reprocessing were correctly performed. No unsafe injection practices were identified.

## Conclusion

The timing of the events and the phylogenetic analysis provide evidence that HCV was transmitted in the GEU. Even though the same endoscope was used in both patients and that parenteral transmission could not be evidenced, the fact that both endoscopies were diagnostic with no mucous rupture nor blood presence, and that endoscope reprocessing is reviewed frequently in this unit, we believe unsafe injection practices were more likely to be responsible for the transmission. Most of recent HCV outbreaks have been attributed to unsafe injection practices.

## Competing interests

None declared

